# Ripretinib-Induced Focal Segmental Glomerulosclerosis in a Patient With Gastrointestinal Stromal Tumor (GIST)

**DOI:** 10.7759/cureus.102513

**Published:** 2026-01-28

**Authors:** Ohm Tripathi

**Affiliations:** 1 Molecular and Cell Biology, University of Connecticut, Mansfield, USA

**Keywords:** chronic kidney disease (ckd), focal segmental glomerulosclerosis, non-diabetic kidney disease, proteinuria, ripretinib

## Abstract

Ripretinib, a multikinase inhibitor, is used to treat refractory forms of advanced gastrointestinal stromal tumors. We describe a case of a 76-year-old male with a history of chronic kidney disease stage 3B/A3 (baseline creatine 2 mg/dL, urine protein/creatinine 3.5 gm/gm), diabetes, and aortic stenosis who presented with a sudden onset of lower extremity edema and weight gain of 20 lbs. Laboratory findings showed acute kidney injury (creatinine 2.5 mg/dL), hypoalbuminemia (albumin 2.4 g/dl), and nephrotic range proteinuria (urine protein/creatinine = 17 gm/gm). Renal biopsy showed focal segmental glomerulosclerosis. Ripretinib was stopped, and he was placed on high-dose steroids. After four months of prednisone, improvement in proteinuria and serum creatinine was seen. The clinical course suggested that podocyte injuries were induced by ripretinib, the disease process was presumptively responded to steroids, and the podocytopathy lasted after discontinuation of the medication.

## Introduction

Gastrointestinal stromal tumor (GIST) is a mesenchymal tumor of the gastrointestinal tract, most commonly located in the stomach and small intestine, but it can develop anywhere throughout the gastrointestinal tract. The majority (80%) of GISTs have activating mutations in the KIT receptor tyrosine kinase gene, and the minority (5-10% ) have activating mutations in the platelet-derived growth factor receptor alpha (PDGFRA) tyrosine kinase gene [1,2]. Ripretinib is a multikinase inhibitor that targets the proto-oncogene KIT and platelet-derived growth factor receptor alpha. It is used to treat patients with advanced and refractory GISTs after failure of other therapies [1,2]. The most common adverse reactions with ripretinib are alopecia and gastrointestinal disturbances [1].

We present a case of FSGS secondary to ripretinib therapy and subsequent treatment with prednisone, highlighting the importance of biopsy in diabetic patients with a sudden increase in proteinuria, the severe systemic effects of ripretinib and prednisone as a potential therapeutic agent.

This article was previously presented as a meeting poster abstract at the 2025 National Kidney Foundation Meeting on April 9, 2025.

## Case presentation

Clinical history and initial laboratory data

A 76-year-old man with a history of diabetes, dyslipidemia, and anemia was evaluated for transcatheter aortic valve replacement and underwent CT angiography as a workup. He was diagnosed with a large abdominal mass, peritoneal nodes, and liver metastasis. He had surgical removal of the mass and the adjacent colon, which led to a diagnosis of GIST. No platelet-derived growth factor (PDGFR) mutation was found. Due to poor response to imatinib mesylate and sunitinib, he was placed on regorafenib, but unfortunately, his disease continued to progress. Subsequently, he was placed on ripretinib. He had presumptive diabetic nephropathy with baseline proteinuria around 2.5-3.5 g (spot urine protein/creatinine gm/gm). Urine protein creatinine remained stable around 2.4 g after taking the ripretinib for three months. 

He lost follow-up to the nephrology clinic and came to the office complaining of 20-pound weight gain and severe lower extremity edema. On examination, he was an obese male, not in distress, and afebrile, with a heart rate of 68 beats/min and blood pressures of 142/88 mmHg. He had normal cardiopulmonary examination findings and 2 plus pitting lower-extremity edema. Laboratory tests are shown in Table 1. HbA1c four months prior to the clinic presentation was 7.2%. A bioprosthetic aortic valve was present without any regurgitation or stenosis on the echocardiogram. The AV mean gradient was 12 mmHg. His proteinuria had worsened to 7.7 gm/gm, but his creatinine was stable at 2.1 mg/dL. At the time, he decided not to pursue the kidney biopsy. He agreed to a kidney biopsy later when his proteinuria continued to worsen (urine PCR 17 gm/gm).

**Table 1:** Laboratory values at presentation

Investigation	Value	Reference range
White blood cells	8.5	3.8–5.3 10⁶/uL
Hemoglobin	9.0	11.0–15.0 g/dL
Hematocrit	29.2	35-46%
Platelets	232	130–400 10^3^/uL
Sodium	140	136–145 mmol/L
Potassium	4.0	3.5–5.1 mmol/L
Chloride	110	98–107 mmol/L
Bicarbonate	16	23–31 mmol/L
Blood urea nitrogen	64	9.8–20.1 mg/dL
Creatinine	2.1	0.57–1.11 mg/dL
Glucose	138	80–115 mg/dL
Calcium	8.1	8.8–10.0 mg/dL
Albumin	3.1	3.2–4.6 g/dL
Magnesium	2.9	1.6–2.6 mg/dL
Hemoglobin A1C	7.2%	4.0-5.6%
Urinalysis	Not available	Not available
Urine protein/creatinine	17 gm/gm	<200 gm/gm

Kidney biopsy

Light microscopy showed two glomeruli with segmental sclerosis and hyalinization of the glomerular tuft. No areas of necrosis, proliferation of the capillary walls, or microthrombi were identified. The interstitium was expanded by moderate interstitial fibrosis with some tubular atrophy involving 30-40% of the renal cortex. There was scattered chronic lymphocytic inflammation in and around the areas of interstitial fibrosis. There was no tubulitis or atypical casts. Mild arteriosclerosis was noted. There was no vasculitis. Immunofluorescence was unremarkable. No glomeruli were available for Electromicroscopyevaluation. Only the renal medulla was identified. Electromicroscopy was deemed not necessary as light microscopy provided the pathological diagnosis, even though electromicroscopy could have added more information about the degree and severity of the foot process effacement to differentiate primary versus secondary FSGS.

**Figure 1:**
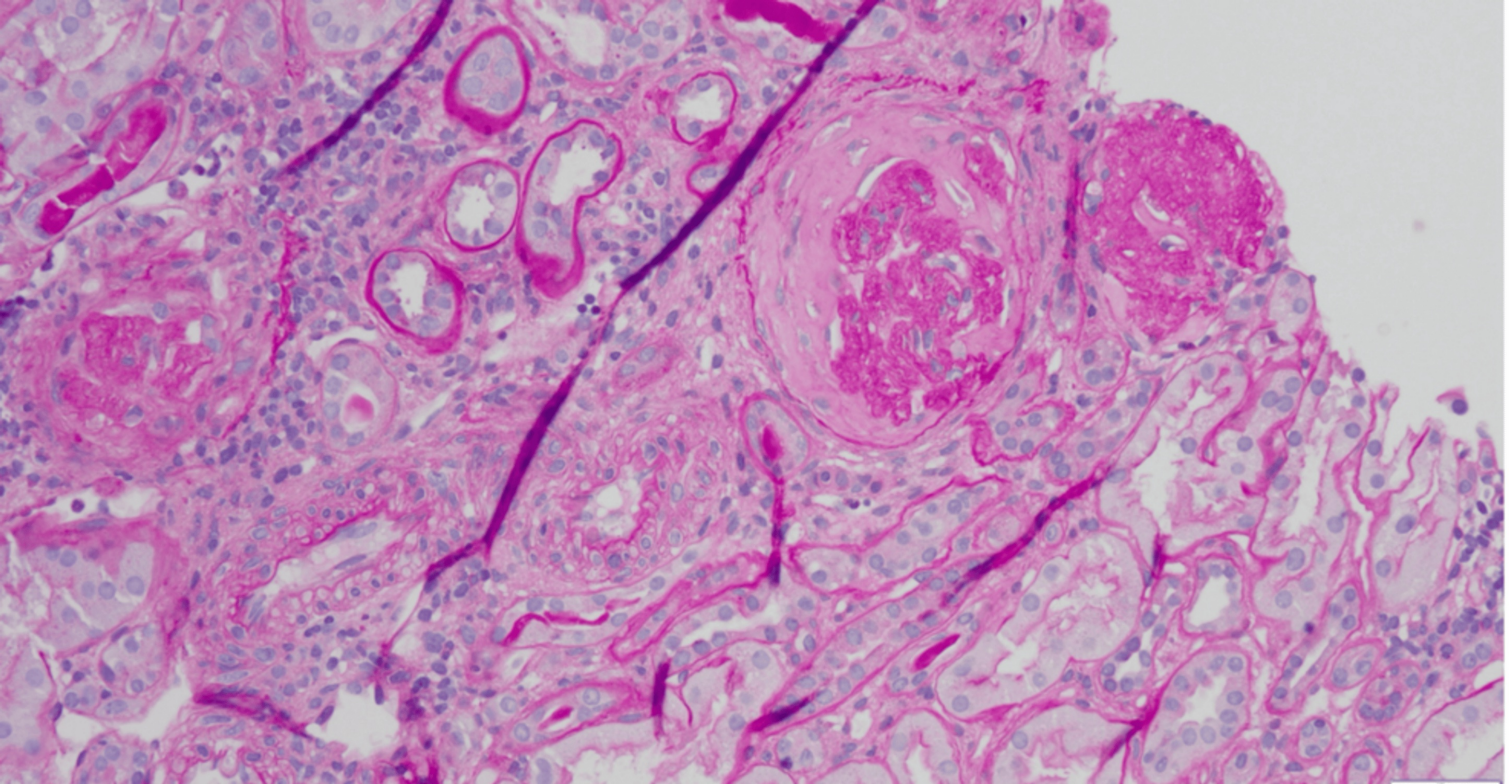
Light microscopy Renal parenchyma revealing cortex and medulla with 5 glomeruli, 2 of which are globally sclerotic. Two glomeruli with segmental sclerosis with hyalinization of the glomerular tuft. Sections are stained with Hematoxylin and eosin, trichrome, and silver. 100x magnification was used.

The final diagnosis was focal segmental glomerulosclerosis, not otherwise specified, with 38% global glomerulosclerosis. Moderate interstitial fibrosis and tubular atrophy involving 30-40% of the renal cortex were seen (Figure 1).

Clinical follow-up

**Figure 2:**
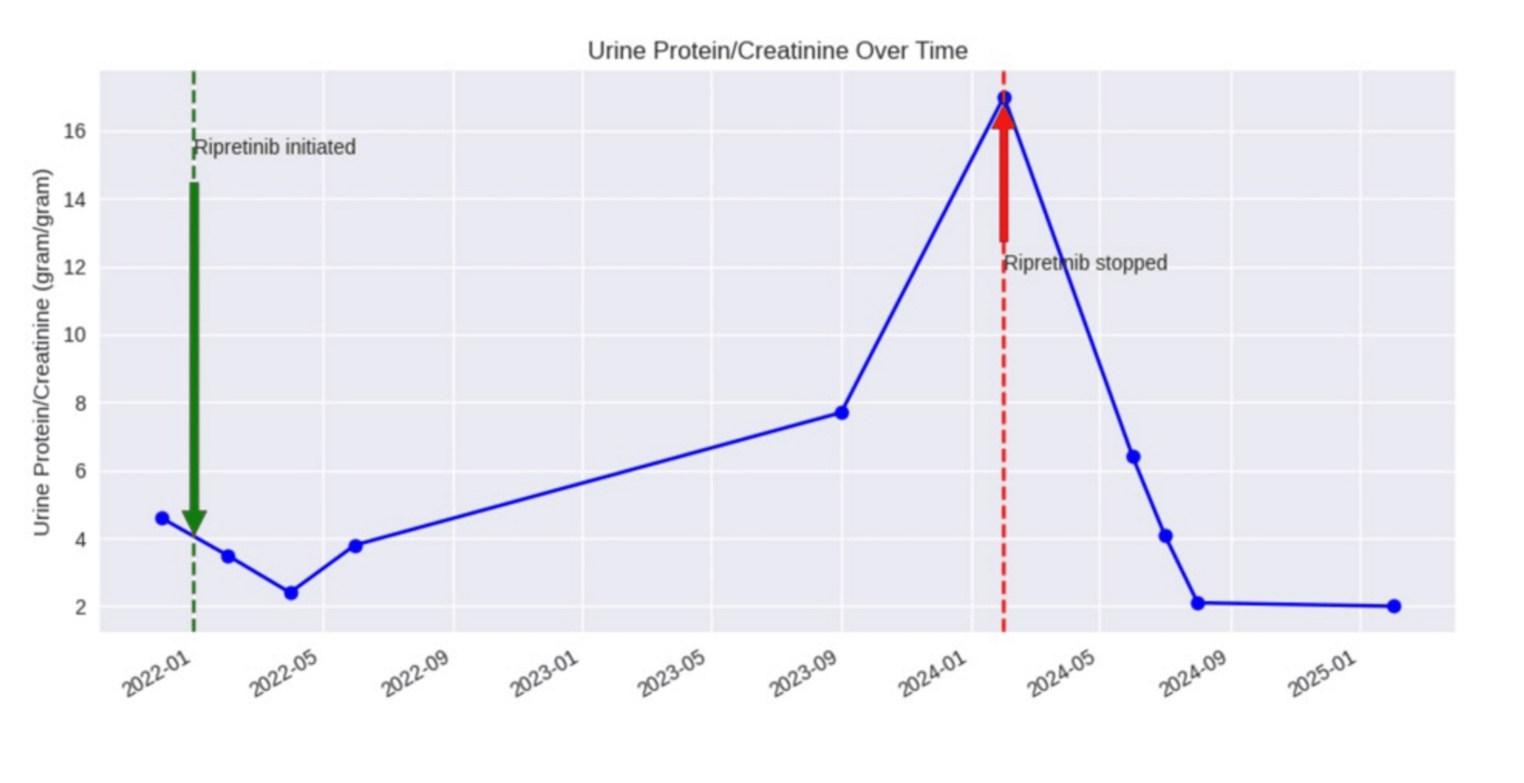
Proteinuria over time Urine protein/creatinine (gram/gram) and relation to ripretinib Image created by the author with OpenAI LLM

Ripretinib was discontinued, and he was placed on empirical oral prednisone (1 mg/kg). After four months of prednisone therapy, proteinuria improved to 4 g and serum creatinine returned to his baseline. He had some improvement in his edema and weight with the help of diuretics. His serum albumin failed to improve. After a lengthy interdisciplinary discussion with his oncology team, the decision was made to resume ripretinib. Proteinuria and renal function remained stable after reintroduction of ripretinib and with continuous use of a small dose of prednisone 10 mg daily (Figure 2).

## Discussion

We report a diabetic patient with GIST who developed worsening proteinuria after ripretinib administration. GISTs are rare tumors of the gastrointestinal tract with mutations in genes that control specific enzymes called tyrosine kinases. Historically, the treatment of GISTs was limited because traditional chemotherapy is ineffective against these tumors. TKIs have extended survival substantially in patients with GIST. However, many GISTs go on to develop secondary mutations that render them resistant to a given TKI. Ripretinib is the fourth drug in the usual treatment sequence recommended for patients with advanced GIST who have progressed (or are treatment intolerant) after receiving three or more TKIs [3].

Worsening baseline proteinuria (3.8-17 gm/gm) and worsening creatinine (2-2.7 mg/dL) occurred after the initiation of ripretinib. Isolated microscopic hematuria, rapid onset of nephrotic syndrome, progressive onset of nephrotic syndrome, rapidly progressive or stepwise reduction in kidney function with relevant proteinuria, and rapidly progressive or stepwise reduction in kidney function with scant proteinuria are some of the indications for the kidney biopsy in a diabetic patient [4]. Due to a sudden increase in proteinuria and declining renal function, a renal biopsy was performed, which showed FSGS. Proteinuria and kidney function responded to holding the ripretinib, and the addition of prednisone might have added benefit.

Imatinib is a highly effective first-line therapy for GIST, but due to the potential for the development of resistant mutations in KIT, some patients require second- and third-line kinase inhibitors such as sunitinib, nilotinib, regorafenib, bosutinib, ponatinib, or avapritinib. Our patient required ripretinib after failing on imatinib, sunitinib, and regorafenib. Ripretinib has been found to have activity in vitro and in vivo against GIST-derived tumor cells with resistant mutations. It has shown to improve progression-free and overall survival in patients with advanced GIST who were refractory to or intolerant of standard kinase inhibitors [5].

To our best knowledge, this is the first case report of FSGS due to ripretinib. As we do not have any data regarding ripretinib and its effect on the kidneys, we have to extrapolate the information available from other TKIs on the kidneys. Focal segmental glomerulosclerosis [6] and Thrombomicroangiopathy [7,8] caused by Imatinib have been reported. Dasatinib and nilotinib can cause nephrotic syndrome. Renal impairment and multiple electrolyte disturbances have also been reported with TKIs [9]. Ripretinib is metabolized in the liver via the cytochrome P450 system, largely CYP3A4 and 2D9. The possible mechanism of injury from ripretinib is unknown. In a meta-analysis of TKIs and their adverse events, ripretinib had lower risks of worsening kidney function, and the incidence of proteinuria was not higher than placebo [10].

We hypothesize that a possible podocyte injury and glomerular microangiopathy might result in glomerulosclerosis. VEGF is present in the podocytes, and it is critical for the development and maintenance of normal glomerular function [11]. VEGF-mediated activation of focal adhesion kinase 1 (FAK) and paxillin requires Src family kinases, which in turn are responsible for cell adhesion and survival. Dasatinib is a potent inhibitor of Src family kinases [12]. Disruption of the VEGF signaling pathway [11] and PDGF inhibition [6] might be one of the possible mechanisms of the kidney injury. Dasatinib inhibition of Src family kinases signaling could possibly lead to disruption of downstream mediators of VEGF signaling and thus proteinuria, as evidenced by lower levels of the phosphorylation of FAK and paxillin, which are the downstream targets of Src kinase, and are lower in the glomeruli in patients treated with dasatinib [13]. Data on the renal recovery from TKIs are mixed, as some studies show partial recovery of renal function after discontinuation [14], while others show no significant recovery [15].

## Conclusions

Our case highlights focal segmental glomerulosclerosis as a clinically significant renal complication associated with ripretinib therapy in a patient with advanced GIST. In diabetic patients with a sudden and marked increase in proteinuria, a kidney biopsy remains essential to distinguish drug-induced glomerular injury from progressive diabetic nephropathy. Just like other TKIs, Ripretinib can potentially cause podocytopathy. Discontinuation of ripretinib and possibly adjunctive corticosteroid therapy led to partial renal recovery, and cautious reintroduction of ripretinib with low-dose prednisone was feasible without further deterioration.
